# *Helicobacter pylori* – HIV co-infection and dyspepsia

**DOI:** 10.1186/1471-2334-14-S4-P44

**Published:** 2014-05-29

**Authors:** Nina-Ioana Șincu, Lucia Carmen Chiriac, Erzsébet Iringó Zaharia-Kézdi, Brînduşa Ţilea, Anca Georgescu, Cristina Gîrbovan, Andrea Incze, Andreea Bodea, Simona Bățagă

**Affiliations:** 1 Department of Infectious Diseases, University of Medicine and Pharmacy Tîrgu Mureş, Romania; 2 Clinic of Infectious Diseases I, Clinical County Hospital Mureş, Romania; 3 Department of Internal Medicine I, University of Medicine and Pharmacy Tîrgu Mureş, Romania

## 

*Helicobacter pylori* infection is one of the most frequent causes of dyspepsia in the general population. As gastro-intestinal symptoms are a common complaint among HIV-positive persons, the purpose of the present study is to assess the role of *Helicobacter pylori* infection in this category of patients.

We performed a retrospective, case-control study on two groups of HIV-infected patients admitted to the Infectious Diseases Clinic I, Tîrgu-Mureş and tested for *Helicobacter pylori* infection by stool antigen detection: group A – 36 dyspeptic subjects and group B – 5 patients without dyspeptic complaints. We compared the two groups from the point of view of the frequency of *Helicobacter pylori* infection. Statistical analysis of data was performed with the help of GraphPad programme.

4 (11.11%) HIV-positive patients with dyspeptic symptoms tested positive for *Helicobacter pylori* infection, while none of the patients in group B was diagnosed with *Helicobacter pylori* infection (p=1.0000, OR=1.523).

Although none of the asymptomatic patients in our study was diagnosed with *Helicobacter pylori*, the absence of a statistically significant association between *Helicobacter pylori* infection and dyspepsia among HIV-positive patients may suggest that other etiologies should be searched in HIV-infected patients with gastro-intestinal complaints.

